# The Correlation between Genotype Richness of Submerged Macrophytes and Periphyton Biomass: A Mesocosm Study Based on Five Dominant Submerged Macrophytes from Yangtze River

**DOI:** 10.3390/plants12132492

**Published:** 2023-06-29

**Authors:** Yu Cao, Xiang-Rong Fan, Henry Kariuki Njeri, Yun-Hai Pu, Wei Li, Yuan-Yuan Chen

**Affiliations:** 1 Aquatic Plant Research Center, Wuhan Botanical Garden, Chinese Academy of Sciences, Wuhan 430074, China; caoyu@wbgcas.cn (Y.C.); fanxiangrong@wbgcas.cn (X.-R.F.); kariukiwestnd@gmail.com (H.K.N.); liwei@lwbgcas.cn (W.L.); 2 Hubei Key Laboratory of Wetland Evolution & Ecological Restoration, Wuhan Botanical Garden, Chinese Academy of Sciences, Wuhan 430074, China; 3 Sino-Africa Joint Research Center, Chinese Academy of Sciences, Wuhan 430074, China; 4 University of Chinese Academy of Sciences, Beijing 101408, China; 5 Wildlife Conservation Station of Hubei Province, Wuhan 430079, China; yunhaipu2002@163.com; 6 Research Center for Ecology, College of Science, Tibet University, Lhasa 850000, China

**Keywords:** genotype richness, *Potamogeton wrightii*, *Hydrilla verticillata*, *Potamogeton macckianus*, *periphyton biomass*

## Abstract

Submerged macrophyte and periphyton are main primary producers which strongly interact with each other in clear water shallow lakes. In this study, the effects of genetic variation of the macrophyte species on periphyton biomass were studied in five submerged species. A two-year mesocosm study was conducted with four levels of genetic diversity (1, 4, 8 and 16 genotypes) for each submerged macrophyte, including 1600 individuals and 320 boxes in 20 mesocosms. Of the five submerged species, only *Vallisneria spinulosa* showed a positive correlation between its levels of genotype richness and the periphyton biomass. The correlation between genetic distance of genotypes and periphyton biomass was tested, which varied with the difference of seasons and species. In summary, we found that in freshwater mesocosms, the genetic diversity of submerged macrophytes may play a role in regulating the periphyton biomass, but the interaction between genetic diversity of macrophytes and periphyton biomass was not straightforward. This study will provide new insights into the interaction dynamics between the two primary producers in shallow lakes.

## 1. Introduction

Submerged macrophytes are principal primary producers in aquatic ecosystems, which can maintain the clear water states through a series of cascading effects, e.g., reducing the sediment re-suspension, competing nutrients with phytoplankton, and providing the refuge and food for zooplankton and small fish [1]. Additionally, periphytic algae are also important primary producers in shallow lakes [2]. Because periphyton attach to the surface of submerged macrophytes, the diversity of morphology of submerged macrophytes greatly affects the community and biomass of periphyton [3,4]. Previous studies about terrestrial plant communities, including forests and grasslands, showed that the genetic variations within foundation trees or herbs produced profound impacts on the abundance and composition of associated communities [5,6,7,8]. For example, Crutsinger et al. [5] established experimental plots that contained genotype gradient of *Solidago altissima*, a perennial herb widely distributed in eastern North America. The experiment showed a positive correlation between the level of genotypic diversity in the herb and the richness and abundance arthropod community. Additionally, based on empirical studies, Bangert et al. [6] discovered that the intraspecific genetic diversity of the foundation species can influence the associated arthropod community and ecosystem processes, using the trees *Populus* spp. from the western North America as the model system. Unfortunately, in aquatic ecosystems, there has been little research on the relationship between the genetic diversity of the foundation species and their related communities. The only such study was about a marine macroalgae *Fucus vesiculosus* from the Northern Baltic Sea, which showed that the genetic diversity of the algae significantly affected the epiphytic community including both algal and invertebrate community [9]. However, this kind of studies with aquatic macrophytes as the foundation species or the host-plant has not been reported in freshwater ecosystems so far. Freshwater lakes not only provide precious resources such as fresh water and aquatic products for human beings, but also have many important ecological functions, including regulating climate, purifying pollution, and protecting biodiversity. Therefore, a new question was raised: Can the genetic diversity of submerged macrophytes affect the periphyton abundance in a freshwater lake?

Understanding the interaction between different levels of diversity is of great help in exploring the mechanism about the maintenance of ecosystem stability in ever-changing environments [10], which will be beneficial to the formulation of management strategy in the ecosystem, especially for the conservation of biodiversity hotspots [11,12]. The Yangtze River Basin is the largest alluvial plain in China, containing thousands of shallow lakes and ponds, and this region is also one of the six global biodiversity hotspots in the large river eco-regions, capable of sustaining a high abundance and richness of aquatic plants [13]. The Honghu Lake is a typical shallow lake and one of the largest lakes in the Yangtze River Basin, with the surface area of over 300 km^2^. The lake has been listed as one of the internationally important wetlands in Ramsar Convention since 2008. In this study, five dominant submerged macrophytes were collected from the Honghu Lake. Our fieldwork has also shown that the five submerged macrophytes were commonly-found species in the other lakes in the Yangtze River Basin. Using these five macrophytes, we established artificial communities with different levels of genotype richness (1, 4, 8 and 16 genotypes) of each macrophyte. The study aimed to investigate the correlation between the genetic diversity of submerged macrophytes and their periphyton biomass. Previous studies showed that the periphyton community was affected by the growth form (e.g., leaf structure and leaf size) or physiological traits (e.g., polarized pH distribution between the two sides of submerged leaves or secretion of allelopathic substances) of host-plants [3,4,9,14,15]. The phenotypic and physiological traits of plants are ultimately determined by the genetic variation of the plants. Therefore, we hypothesized that higher genotype richness in the submerged macrophyte population would form more structurally or physiologically heterogenous environments on the plant surface, which might stimulate the periphyton growth and promote the periphyton biomass. Given that both submerged macrophytes and periphytic algae are primary producers in shallow lake ecosystems [2], this study will provide new insights into the interaction dynamics between the two primary producers, which will benefit the management and restoration of shallow lakes in the Yangtze River Basin.

## 2. Result

### 2.1. The Effects of Macrophytes Species on Periphyton Biomass

**Figure 1:**
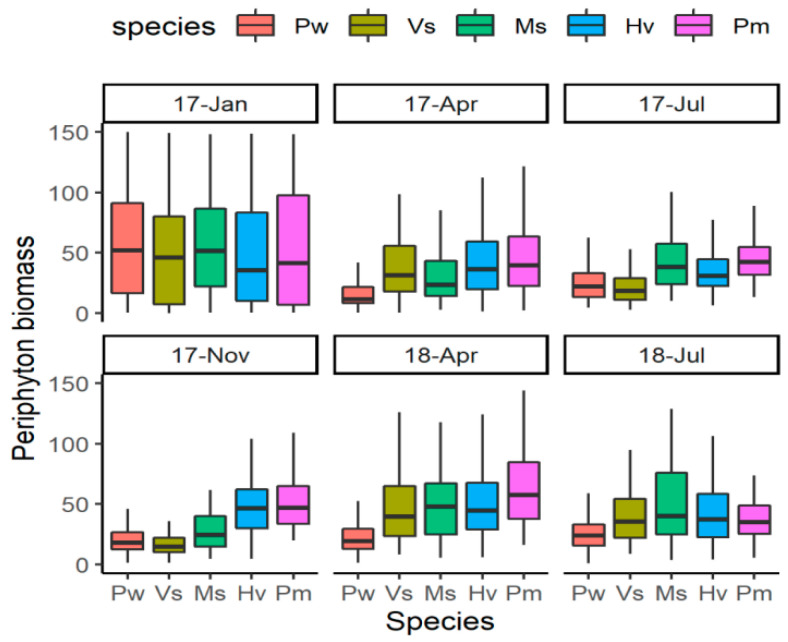
The periphyton biomass on five submerged macrophytes during the experiment. For species name, Pw means *Potamogeton wrightii*; Vs means *Vallisneria spinulosa*; Ms means *Myriophyllum spicatum*; Hv means *Hydrilla verticillata*; Pm means *P. macckianus*. The dates (17-Jan, 17-Apr, 17-Jul, 17-Nov, 18-Apr, 18-Jul) were shorten as Year-abbreviation of Month for January 2017, April 2017, July 2017, November 2017, April 2018 and July 2018.

In general, the periphyton biomass was low, the mean values being <100 ug Chl dw ^−1^. During the two-year experimental period, there were significant differences among the five species (LMM, *F* = 71.13, *p* < 0.001). Compared with that on other two species *Myriophyllum spicatum* and *Vallisneria spinulosa*, the periphyton biomass on *Potamogeton wrightii* was significantly lower and on *P. macckianus* was higher (Figure 1). *Hydrilla verticillata* had a significantly higher periphyton biomass than *P. wrightii* and *V. spinulosa,* but was not different from the rest two species.

### 2.2. Effects of Genotype Richness on Periphyton Biomass for Each Species

**Figure 2:**
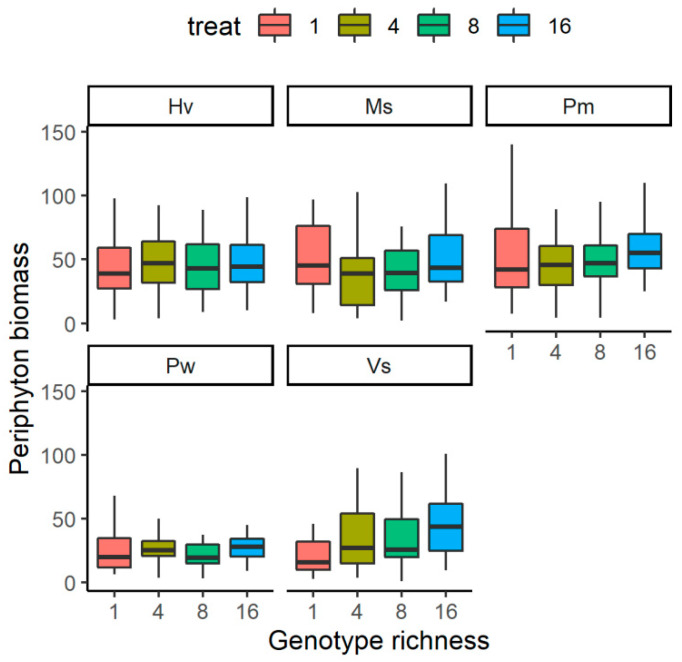
The effects of genotype richness levels on periphyton biomass growing on each species. For species name, Pw means *Potamogeton wrightii*; Vs means *Vallisneria spinulosa*; Ms means *Myriophyllum spicatum*; Hv means *Hydrilla verticillata*; Pm means *P. macckianus*.

The relationship between the genotype richness levels of submerged plants and the periphyton biomass was species-specific (Figure 2). The periphyton biomass on *V. spinulosa* showed an increasing trend along with the increase of genotype richness levels; it was significantly higher in G16 than in G1 (*F* = 5.622, *p* < 0.01), but it did not differ between the two treatments with the left two. For the rest four species, it did not differ significantly among the four genotype richness levels (*F* < 2.47, *p* > 0.05 for all). 

### 2.3. Correlation Analysis between the Genetic Distance and the Difference of Periphyton Biomass for Different Genotypes

**Figure 3:**
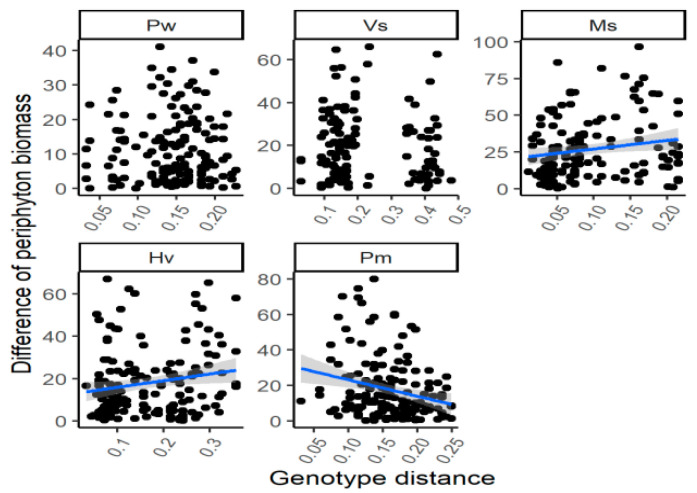
The correlations between the genetic distance and the differences of periphyton biomass of the individuals with different genotypes by the end of the experiment. For species name, Pw means *Potamogeton wrightii*; Vs means *Vallisneria spinulosa*; Ms means *Myriophyllum spicatum*; Hv means *Hydrilla verticillata*; Pm means *P. macckianus*.

There was no significant relationship between the genetic distance and the difference of periphyton biomass for the individuals with different genotypes for the species *V. spinulosa* throughout the experimental period (Table 1; Figure 3). However, the genetic distance was (marginally) positively correlated with the variation of periphyton biomass for *P. wrightii* in July 2017 and for *H. verticillata* (*p* = 0.060) and *M. spicatum* (*p* = 0.054) in July 2018. Besides, the negative relationship between the genetic distance and the difference of periphyton biomass among different genotypes was found for *P. macckianus* in April 2017, July 2017 and July 2018 and for *H. verticillata* in January 2017 (for all four correlation, *p* < 0.05). 

## 3. Discussion

Previous studies elucidated that the periphyton biomass was influenced by some abiotic factors, like light availability (or transparency) and nutrient supply in the water column [16,17]. In this study, the water in mesocosms was of bottom-view transparency, and macrophytes grew in the similar nutritional state within one mesocosm, which did not support the effects on periphyton biomass from these factors. This research evaluated the effects of biotic factors (host plant) of the submerged macrophytes on periphyton biomass and found the species-specific responses of periphyton to different host (i.e., submerged macrophytes). To our knowledge, this is the first attempt to investigate the relationship between the genetic diversity of macrophytes and their related communities in aquatic ecosystems. 

### 3.1. Effects of Genotype Richness on Periphyton Biomass

Among the five submerged macrophytes tested, *V. spinulosa* showed higher periphyton biomass with increasing genotype numbers of the plant. Similarly, previous studies from the terrestrial ecological system showed that host-plant genotypic diversity influenced the structure and abundance of associated communities [5,18]. For example, aboveground primary production of terrestrial herbaceous *Solidago altissima*, together with the abundance of arthropods, herbivores and related predators, increased with the plant genotype numbers from 1, 3, 6 to 12. This was attributed to the increasing intraspecific genotypic diversity of the host-plant, which increased the diversity of resources available to associated communities [5]. The exclusive study in aquatic ecosystem showed that the different genotypes of marine algae (*F. vesiculosus*) had significantly different epiphytic and invertebrate communities. Additionally, the study also found that neither neutral genetic markers nor a single phenotypic trait could provide a mechanistic understanding of the genetic basis of community [9]. 

In the present study, the failure of such tendency in the other four submerged species may result from the distinct physiological traits of macrophytes which overruled the effects of genotype diversity. For instance, the polarized leaf of *P. wrightii* induced different pH values between the two sides of the leaves, which could lead to a lower species richness and diversity of periphytic algae on nearby substrate [4]; *M. spicatum* released allelopathic chemicals which inhibited periphyton growth [19]. Therefore, as recommended by the previous study [9], more quantitative trait variations are needed to comprehensively understand the underlying mechanism. Alternatively, although the genetic diversity of foundation trees/herbs was correlated with species diversity and richness of associated communities, such correlations showed space and time scale-dependent (more details were discussed in the cited review [6]). In the present study, all macrophytes were originally collected from different regions of the Honghu Lake in the middle and lower reaches of the Yangtze River. The water depth, sediment and light conditions vary greatly in different regions of the large shallow lake [20]. Additionally, the duration of this experiment is two years (from August 2016 to July 2018). For the four macrophytes (*P. wrightii*, *P. maackinaus*, *M. spicatum* and *H. verticillata*), the spatial and temporal scale of this study might have exceeded the applicable scope of the correlation, which also might lead to no correlation between the genotypic diversity of these four plants and the periphyton communities.

In summary, there were species-specific responses of genotype richness levels on periphyton biomass. Furthermore, in contrast to our hypothesis, the promoting effects of higher genotype richness on periphyton growth were not universal for all species.

### 3.2. Correlations between Genetic Distance for Genotypes and Periphyton Biomass

The positive correlations between plant genetic distance and population production have generally been documented in terrestrial ecosystems [11,12]. However, in the present study, only few consistent cases were found, e.g., in July 2017 for *P. wrightii*. In contrast, no significant correlation was found between the variation of periphyton biomass for *V. spinulosa* and its genetic distance of genotypes. The previous study of marine ecosystem also failed to show significant correlations between the variation of epiphytic algae on the marine algae and genetic distance between different genotypes [9]. Given that *V. spinulosa* has a very simple strap-like leaf type, its leaf growth (i.e., elongation) would strongly dilute the accumulation of periphyton [14]. Combining with results that the higher periphyton biomass in G16 than in G1 for *V. spinulosa*, it is argued that, instead of neutral genetic markers, the quantitative trait variations (such as leaf growth rates) might result in the significant differences among different genotypes, as assumed in the previous study [9]. The genetic marker diversity of host-plants might not directly reflect the diversity of morphological and physiological traits which can affect periphyton growth and biomass accumulation. 

### 3.3. Implications for Lake Restoration

Aquatic plants and their periphytic algae are two basic producers in freshwater ecosystems, and they play a vital role in maintaining the stability of freshwater ecosystems. Thus, understanding the effects of common aquatic plants on their periphytic algae biomass will be beneficial for making the lake management and restoration strategies. Combining with previous studies [3,4,15,19], we recommended that leaf structures and plant genotypes should be considered simultaneously when transplanting common aquatic plants to restore lakes. The specific recommendations are as follows: Firstly, the periphyton biomass accumulated easily on submerged macrophytes with complex leaf structures [3,15]. This was also proved in the present study. Therefore, macrophytes with simple leaf structure are good candidates for lake restoration when periphyton biomass was expected to be high. Secondly, *V. spinulosa* showed positive correlation between periphyton biomass between different levels of genotype richness during the experiment. Given that macrophytes of the genus *Vallisneria* are one of the most popular species of macrophyte community restoration in China, their genotype richness should be considered when used for lake restoration. Additionally, plant physiological processes, like the polarized leaf of *P. wrightii* and allelopathic chemicals released by *M. spicatum*, also put important impacts on the accumulation of periphyton [4,19]. Therefore, when using macrophytes to regulate periphyton, the species-specific physiological traits of the plants also should be considered.

## 4. Materials and Methods

### 4.1. Species Selection 

**Figure 4:**
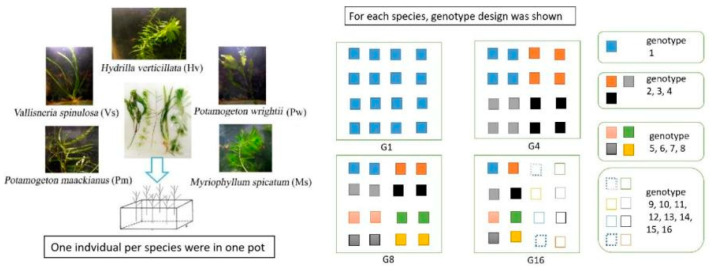
Diagram of the experimental design showing the structure of five submerged macrophytes and the arrangement of each genotype. The pots were placed randomly in the ponds. To help understand our experiment design, the randomness was not explicitly shown in the figure.

Based on years of fieldwork in the Yangtze River Basin, we selected five dominant submerged macrophytes in shallow lakes for the establishment of experimental community, and the macrophytes included *P. wrightii*, *P. maackinaus*, *M. spicatum*, *V. spinulosa* and *H. verticillata* (Figure 4). All the macrophyte materials were collected in 54 sites in the Honghu Lake (E 113°12′–113°26′, N 29°40′–29°58′), a typical shallow lake in the Yangtze River Basin. The 54 sampling sites were identified based on Thiessen polygons described by Li et al. [21]. For each species, more than 20 individuals were sampled at intervals of at least 10 m to avoid collecting the same individual. All plant were immediately brought back to the laboratory and planted in experimental pools for further use.

### 4.2. DNA Extraction and Genetic Analysis

AFLP (amplified fragment length polymorphism) analysis was used to identify the genotypes of each submerged macrophyte because of its high stability and polymorphism. For each individual, total genomic DNA was extracted from 0.6 g fresh leaves using the CTAB (cetyltrimethylammonium bromide) method described by Doyle and Doyle [22]. The AFLP protocol was essentially modified from Vos et al. [23] and performed as described by Li et al. [24]. The productions of selective PCR were resolved on 6% urea-denatured polyacrylamide gels and visualized by silver staining. The unambiguous polymorphic fragments (50–500 bp) were scored as present (1) or absent (0), and the binary matrix was constructed for further analysis. The software GenoType/GenoDive was employed to test whether the individuals had been collected twice [25]. Using the software GenAlEx 6.5 [26], we determined 16 genotypes for each species which were used in the following experiment design. For each species, the genetic distance among the 16 genotypes was measured by POPGENE 1.31 [27] and the results were shown in Figure S1 in the Supplementary Materials.

### 4.3. Experiment Treatment-Genotype Richness Levels

The mesocosm study was conducted in the Wuhan Botanical Garden (E 114°24′, N 30°32′). After confirming the genetic genotypes of the five submerged macrophytes, we conducted the clonal cultivation for each genotype of the five submerged species. After one-year pre-cultivation, we obtained enough ramets for each genotype, and initiated the mesocosm study on 16 August 2016. 

Four levels of genotype richness (1, 4, 8 and 16 genotypes) were constructed for each species. The genotypes appearing in treatments with low-level genotypes richness were nested in treatments with high levels of genotypes (e.g., all the four genotypes used in 4-genotype treatment were included in the 8-genotype treatment), and it referred to G1, G4, G8 and G16 hereafter (details shown in Figure 4). Each pond (size of length × width × height: 2 m × 2 m × 1 m) was planted with 80 individuals (ca. 10 cm length) in 16 plastic pots with an equally spaced 4 × 4 grid. Each pot (size of length × width × height: 40 cm × 30 cm × 15 cm) was filled with 10 cm depth of sediment, and cultivated with five individuals, with one for each species. For G1, 16 individuals from the same clone of each species were planted; for G4, 16 individuals with four genotypes (including the genotype used in G1) of each species were planted, and every four individuals shared one genotype; for G8, 16 individuals with eight genotypes (including the four genotypes used in G4) of each species were cultivated, and every two individuals shared one genotype; for G16, 16 individuals with 16 genotypes (including the eight genotypes used in G8) of each species were planted. Each genotype richness level consisted of five mesocosms as replicates. In summary, 1600 macrophyte individuals were transplanted and cultivated in 20 ponds.

The water in the mesocosm was originally from the nearby Donghu Lake, and the water level was maintained at 1 m by adding the tap water monthly. The added nutrient from the tap water was negligible based on the previous studies [28,29]. The nutrient levels were low (total nitrogen ranging within 0.05~0.53 mg L^−1^ and total phosphorus ranging within 3~63 μg L^−1^) and low phytoplankton biomass (ranging within 0.3~15.4 μg L^−1^). The water transparency was clear to the bottom throughout the experiment.

The periphyton was sampled, respectively, in January 2017, April 2017, July 2017, November 2017, April 2018 and July 2018 (abbreviated as Year-abbreviation of Month such as 17-Jan, 17-Apr, 17-Jul, 17- Nov, 18- Apr, 18-Jul). According to the approach described by Cao et al. [16], we carefully harvested the third leaf of *P. wrightii* and *V. spinulosa*, and the top-10 cm shoot tip for *M. spicatum*, *H. verticillata* and *P. maackianus*. The leaves/tips were placed in plastic bags and stored in a cooling box at 4 °C until the periphyton collecting. As for every sampling event, all genotypes and species in four genotype richness levels were sampled. In the laboratory, the plastic bags were fiercely shaken for one minute. Then, the leaves/tips were taken out from the bags and placed in plastic trays, and the plant materials were carefully brushed with soft brushes. All the experimental items, including the leaves/tips, plastic bags, trays and brushes, were rinsed three times with tap water. All the wash-offs were filtered through a GF/C filter for chlorophyll a determination after ethanol extraction, and the determined chlorophyll a content was recorded as periphyton biomass [16].

### 4.4. Data Analysis

The raw data were visually inspected. Then, the extreme outliers (larger than 3 times of the interquartile range) were identified and removed by using the function ’boxplot’ in R 4.1.2 [30]. The residuals of each model were visually checked to ensure the homogeneity and normal distribution of data. If needed, the data was log transformed. As the experiment progressed, some individuals died. Given the missing of certain genotype (more details in Supplementary Materials: data_number.xlsx), only the treatment with enough replicates (*n* > 3) was included in the following data analysis.

A global linear mixed model (LMM) were used (fit_species <- lmerTest::lmer (peri_biomass ~ species + treat + (1|Genotype) + (1|SamplingTime), data = data_final)), with the macrophyte species and genotype richness (treat) as the fixed effects, and the genotype of each individual and the sampling time as the random effects [31]. A *post-hoc* test was conducted in the package ‘multcomp’ by setting ‘species’ as the indicator for multiple comparison, respectively. For the different effects of genotype richness on each species, linear mixed models (fit_genotype <- lmerTest::lmer (peri_biomass ~ treat + (1|SamplingTime), data = data_sp)) were further used to compare the difference of periphyton biomass by setting sampling time as the random factor and genotype richness (‘treat’ in the model) as the fixed factor for each species separately. 

To test the effect of genetic variation between macrophyte genotypes on periphyton biomass for each species, two methods were used. Firstly, Cohen’f was used as the indicator of the effect size of the genotype richness treatment on the variation of the periphyton biomass. A rule of thumb for Cohen’f is that 0.1~0.25 means small effects, 0.25~0.4 means medium effect and >0.4 means large effect [32]. Cohen’f was calculated based on the function cohen’f in the package ‘sjstats’. Most data showed a large (at least medium) effect size for each genotype treatment (G4, G8 and G16, respectively) throughout the experiment (details in Figure S2 in the Supplementary Materials). Thus, secondly, we calculated the genetic distance between the genotypes for each species, and then calculated the difference of periphyton biomass between these genotypes. The correlation between the two was analysed, and the focus here was to find whether there was closely linked relationship between the genetic distance between these macrophyte genotypes and the differences of periphyton biomass on these macrophyte genotypes.

## 5. Conclusions

Our results indicated that higher genotype richness can promote the periphyton biomass, and genetic distance between different genotypes can correlate with the difference of periphyton biomass between these genotypes. However, these results were not consistent within all five chosen macrophyte species. In summary, our study documented that in freshwater ecosystems, the genetic diversity of submerged macrophytes played a complex role in regulating the periphyton biomass, but the interaction between genetic diversity and periphyton biomass was species-specific and with changes at different growing periods (i.e., seasonality).

## Figures and Tables

**Table 1:** The statistical summary (correlation coefficient R and the significant level *p*) of the correlation analysis between the genetic distance and the difference of periphyton biomass between the genotypes. Date is shortened as year-abbreviation of month (e.g., 17-Jan means January of 2017). For species name, Pw means *Potamogeton wrightii*; Vs means *Vallisneria spinulosa*; Ms means *Myriophyllum spicatum*; Hv means *Hydrilla verticillata*; Pm means *P. macckianus*. For the significant level, NS means *p* > 0.05; * means *p* < 0.05; ** means *p* < 0.01.

Date	Pw	Vs	Ms	Hv	Pm
17-Jan	−0.074, NS	−0.093, NS	−0.053, NS	−0.220, **	−0.063, NS
17-Apr	0.068, NS	−0.160, NS	−0.120, NS	−0.130, NS	−0.180, *
17-Jul	0.170, *	−0.060, NS	−0.026, NS	0.077, NS	−0.160, *
17-Nov	0.059, NS	−0.008, NS	−0.100, NS	−0.067, NS	−0.060, NS
18-Apr	0.120, NS	0.024, NS	−0.041, NS	−0.023, NS	0.0025, NS
18-Jul	0.0017, NS	−0.080, NS	0.150, *p* = 0.05	0.14, *p* = 0.06	−0.240, *

## Data Availability

Information of Data was provided in the Supplementary Materials.

## Supplementary Materials

The following supporting information can be downloaded at: https://www.mdpi.com/article/10.3390/plants12132492/s1, Figure S1: The statistical distribution of genetic distance of five submerged species based on Nei (1978). Figure S2: The effect size of genotype richness on the variation of periphyton biomass on five macrophytes for each sampling dates.
