# Supplementary material for: The Correlation between Genotype Richness of Submerged Macrophytes and Periphyton Biomass: A Mesocosm Study Based on Five Dominant Submerged Macrophytes from Yangtze River
Source: Plants (Basel). 2023 Jun 29;12(13):2492. doi: 10.3390/plants12132492 (PMC10346411; doi:10.3390/plants12132492)
Supplement: Supplementary file 1 [file plants-12-02492-s001.zip › plants-2413467-supplementary.pdf]

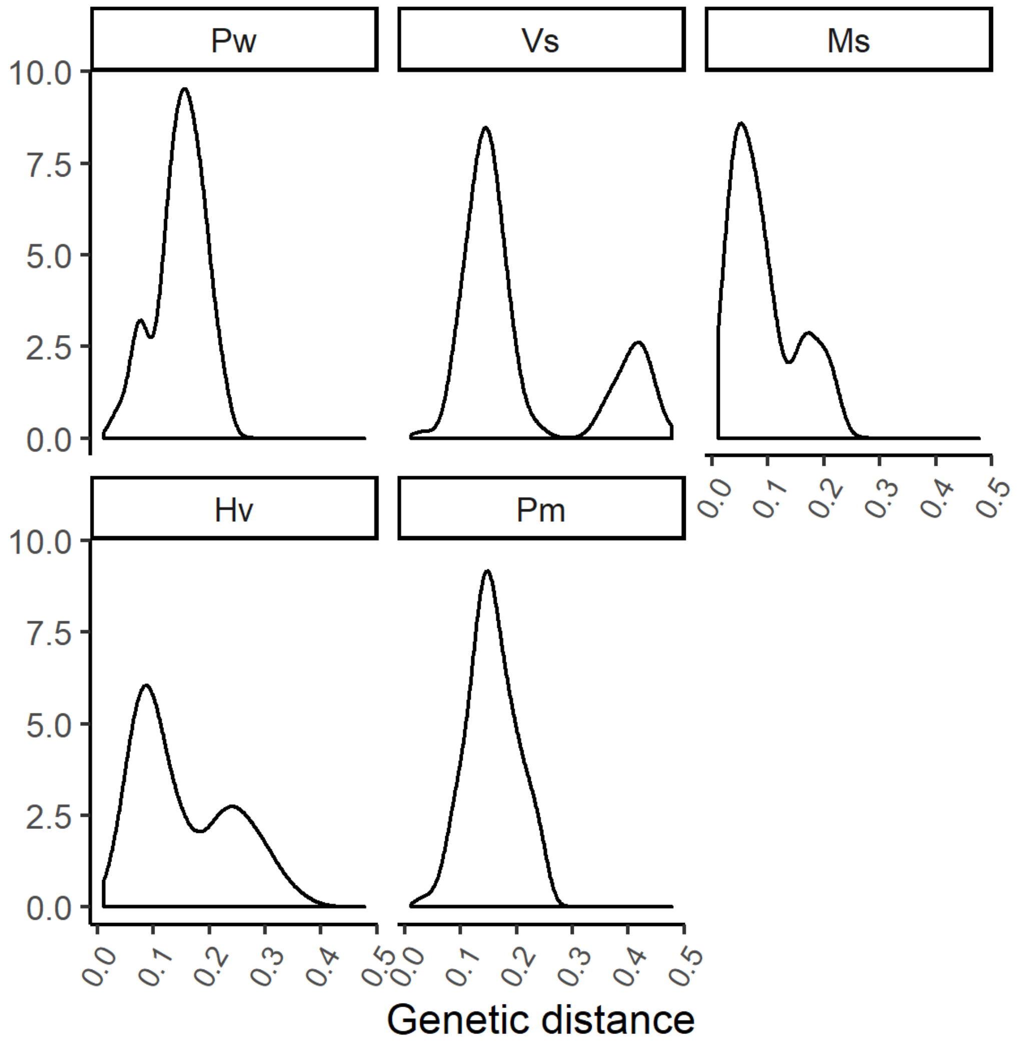

Figure S1 The statistical distribution of genetic distance of five submerged species based on Nei (1978). For species name, Pw means *Potamogeton wrightii*; Vs means *Vallisneria spinulosa*; Ms means *Myriophyllum spicatum*; Hv means *Hydrilla verticillata*; Pm means *P. macckianus*.

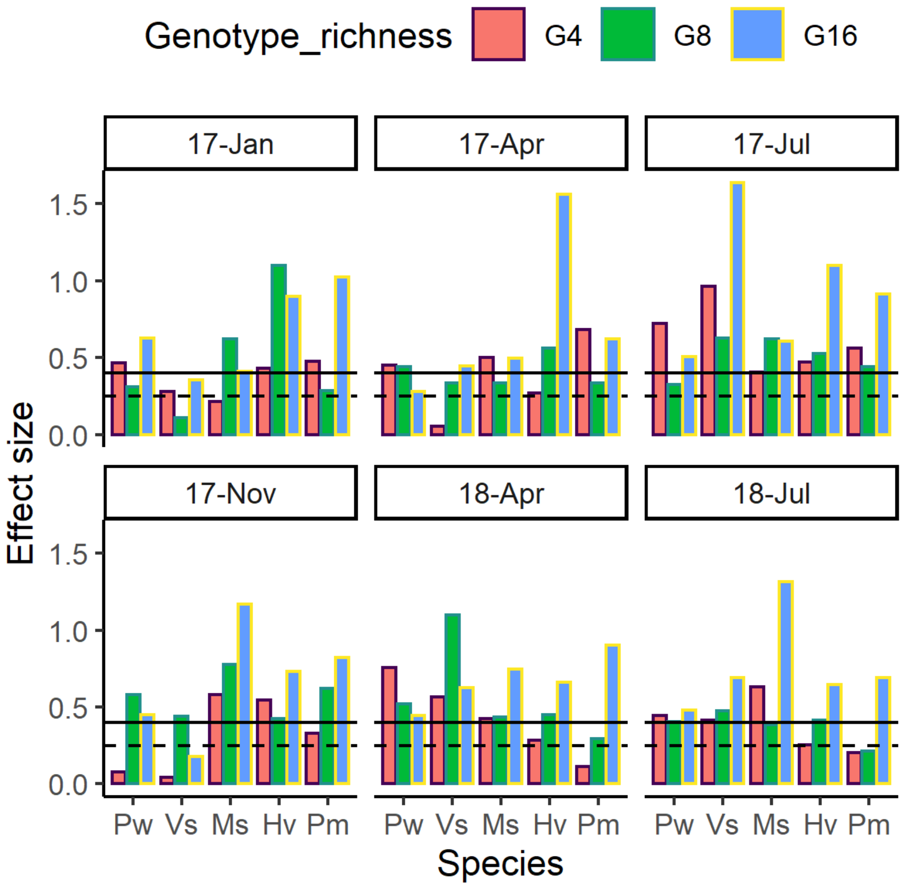

Figure S2 The effect size of genotype richness on the variation of periphyton biomass on five macrophytes for each sampling dates. The solid and dashed line indicates a large and medium effect size, respectively. For species name, Pw means *Potamogeton wrightii*; Vs means *Vallisneria spinulosa*; Ms means *Myriophyllum spicatum*; Hv means *Hydrilla verticillata*; Pm means *P. macckianus*. The dates (17-Jan, 17-Apr, 17-Jul, 17- Nov, 18- Apr, 18-Jul) were shorten as Year-abbreviation of Month for January 2017, April 2017, July 2017, November 2017, April 2018 and July 2018.
